# Drug-target and disease networks: polypharmacology in the post-genomic era

**DOI:** 10.1186/2193-9616-1-17

**Published:** 2013-12-05

**Authors:** Ali Masoudi-Nejad, Zaynab Mousavian, Joseph H Bozorgmehr

**Affiliations:** Laboratory of Systems Biology and Bioinformatics (LBB), Institute of Biochemistry and Biophysics, University of Tehran, Tehran, Iran

## Abstract

With the growing understanding of complex diseases, the focus of drug discovery has shifted away from the well-accepted “one target, one drug” model, to a new “multi-target, multi-drug” model, aimed at systemically modulating multiple targets. Identification of the interaction between drugs and target proteins plays an important role in genomic drug discovery, in order to discover new drugs or novel targets for existing drugs. Due to the laborious and costly experimental process of drug-target interaction prediction, *in silico* prediction could be an efficient way of providing useful information in supporting experimental interaction data. An important notion that has emerged in post-genomic drug discovery is that the large-scale integration of genomic, proteomic, signaling and metabolomic data can allow us to construct complex networks of the cell that would provide us with a new framework for understanding the molecular basis of physiological or pathophysiological states. An emerging paradigm of polypharmacology in the post-genomic era is that drug, target and disease spaces can be correlated to study the effect of drugs on different spaces and their interrelationships can be exploited for designing drugs or cocktails which can effectively target one or more disease states. The future goal, therefore, is to create a computational platform that integrates genome-scale metabolic pathway, protein–protein interaction networks, gene transcriptional analysis in order to build a comprehensive network for multi-target multi-drug discovery.

## Purpose

Identification of the interaction between drugs and target proteins plays an important role in genomic drug discovery, in order to discover new drugs or novel targets for existing drugs. Due to the laborious and costly experimental process of drug-target interaction prediction, *in silico* prediction could be an efficient way of providing useful information in supporting experimental interaction data. Since, a small number of experimental drug-target interaction data has been reported in current and publicly available databases, this has motivated many researchers to develop high performance computational approaches capable of detecting new pairs of drug-target interaction efficiently.

Drug-target network (DTN) is a bipartite graph in which every link connects a drug to a protein if the protein is a known target of the drug (Yildirim et al. 2007). To generate a DT network, all FDA-approved drugs and their known targets are used and the information about drug-target interactions could be extracted from known databases, including KEGG DRUG (Goto et al. 2002), DrugBank (Wishart et al. 2008) and others.

Yildirim et al. released some significant features related to the network topology of the DT network by applying network analysis to drugs and target proteins. Based on their findings, the membrane proteins are mostly targets of FDA-approved drugs which belong to the same Anatomical Therapeutic Chemical (ATC) class that naturally target the same proteins. To investigate the relationships between approved drugs, they integrated all available DT interaction data along with genetic-disease associations, gene expression and protein-protein interaction data.

## Related works

A variety of computational methods have been proposed to analyze and detect new protein-ligand interactions. To the best of our knowledge, traditional computational approaches can be categorized in three classes, namely ligand-based, target-based, and text-mining methods. The Ligand-based approach like QSAR (Quantitative Structure Activity Relationship) uses machine learning methods to predict protein-ligand interaction by comparing a new ligand to the known ligands of a target protein (Butina et al. 2002; Byvatov et al. 2003). When the number of known ligands for a target protein of interest is insufficient, this approach couldn’t be effective in the prediction of interaction. Target-based approach or docking simulation rely on the 3D structure of proteins to predict protein-ligand interaction and it can’t be applied to proteins with unknown 3D structure (Cheng et al. 2007; Donald 2011; Morris et al. 2009). This limitation is very serious for membrane proteins such as Ion channels and G-Protein Coupled Receptors (GPCRs) due to the complexity of determining 3D structures of most of these proteins. Another approach involves text mining methods which are based on keyword searching in literatures but the redundancy in the name of the gene/compound in the literatures is a major concern in this approach (Zhu et al. 2005).

To predict the drug-target interaction, another interesting approach was proposed by Campillos et al. based on the side-effect similarities between known drugs (Campillos et al. 2008). Nevertheless, this approach has been verified by *in vitro* experiments in some cases, it can only apply to the marketed drugs with known side effects and to the interaction between new drugs cannot be predicted by this approach. Recently, the importance of chemogenomic approaches in the domain of protein-ligand interaction prediction has grown fast (Dobson 2004; Kanehisa et al. 2006; Stockwell 2000). These methods integrate both genomic spaces of target proteins, and chemical space of compounds, to predict new drug-target pairs. A unified space, namely “pharmacological space”, could be created by the integration of drug chemical structures, protein sequences and drug-target network topology to infer unknown drug-target interactions. The underlying idea is that drugs with similar chemical structures are likely to interact with similar proteins, and the prediction could be performed by extracting different features for drugs and proteins to define the similarity between two compounds or two proteins.

Based on this concept, a variety of statistical and learning methods have been developed to predict drug-target interaction (Bleakley and Yamanishi 2009; Chen et al. 2012; Cheng et al. 2012; Mei et al. 2012; van Laarhoven et al. 2011; Xia et al. 2010; Xie et al. 2012; Yamanishi et al. 2008). Although promising results have been gained by using these methods, most of them can only predict whether a drug interacts with a target protein, but reveal no further information about how this interaction occurred. Unlike these methods, Wang et al. have proposed a method based on the restricted Boltzmann machine to predict different types of interaction between drugs and targets (Wang and Zeng 2013). This state-of-the-art technique involves research that takes into account the drug-target network in their analyses. “Network pharmacology” or “Systems pharmacology” is therefore a possible next paradigm in drug discovery which is generated by the advances in these areas (Hopkins 2007,2008).

## Discussion

The improvements in drug discovery for complex diseases could be achieved by studying drug action through the network biology. With the growing understanding of complex diseases, the focus of drug discovery has shifted away from the well-accepted “one target, one drug” model, to a new “multi-target, multi-drug” model, aimed at systemically modulating multiple targets. In this context, polypharmacology has emerged as a new paradigm to overcome the recent decline in productivity of pharmaceutical research (Wermuth 2004). Drug designers traditionally consider the polypharamacology as an unwanted property that must be removed or reduced to produce drugs that interact with a single target. But in the modern pharmaceutical industry, the hitherto strategy of, “one drug for one target for one disease”, has been considered responsible for the more-funding-less-drug problem. Recent research has shown that effective treatments of complex diseases are not possible by interventions at single nodes. To modify phenotypes, it is required that multiple proteins be modulated simultaneously. Thus, drugs which act on two or more targets of interest should be more impressive than single-target ones. Assessing the role of polypharmacology in drug action through drug-target network analysis may provide insights into which drugs are more efficacious in the treatment of complex diseases.

The integration of drug-target network with the human disease network revealed that drug targets are often involved in multiple diseases. Another analysis of the OMIM database of genetic associations manifest that most disease shares the genetic origins with others. According to these findings, drug repositioning has been growing in importance in the last few years for the application of known drugs in new directions. In addition, the mapping of drug targets on to the human protein interaction network reveals that the drug targets tend to be highly connected. Thus, the integration of drug-target network with other biological networks can help with drug discovery in producing drugs with more efficacies and less toxicity. Recent research also indicates that besides primary network parameters, more complex network metrics such as motifs and clusters may also be appropriate parameters for controlling the metabolic systems. Moreover, for metabolic networks, enzyme-centric networks could be more reliable in the context of controllability, although little attention has been paid to such networks in systems controllability. The outcomes of metabolic network controllability could create insights into the discovery of novel drug targets (Asgari et al. 2013).

**Figure 1:**
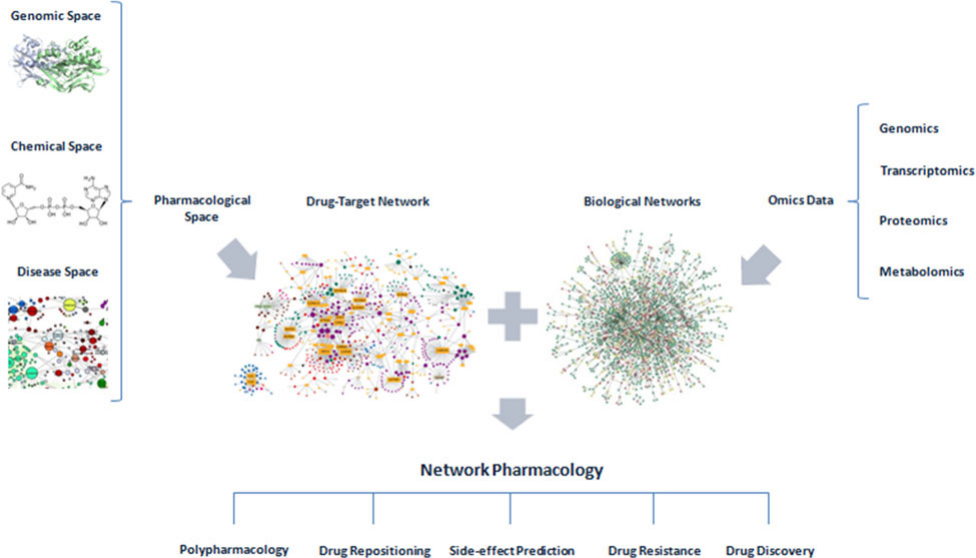
**Polypharmacology in the post-genomic era using pharmacological space.**

Drug discovery also can be influenced by the exploitation of “omics” data. Due to the advances in genomics, transcriptomics, proteomics and metabolomics, large amounts of data have been provided on drug-target interactions. To speed up the drug development process, the information flow must be analyzed by more effective tools in the early stages of drug discovery pipeline. The emerging field of “systems pharmacology”, which relies on “omics” technologies, can explain both therapeutic and adverse effects of drugs through analyses at multiple scales of biological networks (Figure 1).

## Conclusions

An important notion that has emerged in post-genomic drug discovery is that the large-scale integration of genomic, proteomic, signaling and metabolomic data can allow us to construct complex networks of the cell that would provide us with a new framework for understanding the molecular basis of physiological or pathophysiological states. Such an integrated view has important implications in improving our understanding of the disease phenotypes by viewing them as perturbations in a complex system rather than as effects on a selective set of proteins. Using such a framework, network based drug discovery aims to harness this knowledge to investigate and understand the impact of interventions, such as candidate drugs, on the molecular networks that define different states and therefore can significantly complement the existing drug discovery pipelines.

An emerging paradigm of polypharmacology in the post-genomic era is that drug, target and disease spaces can be correlated to study the effect of drugs on different spaces and their interrelationships can be exploited for designing drugs or cocktails which can effectively target one or more disease states (Janga and Tzakos 2009). This understanding could lead to the introduction of new multidrug treatments, side-effect prediction and the identification of new drug targets. The future goal, therefore, is to create a computational platform that integrates genome-scale metabolic pathway, protein–protein interaction networks, gene transcriptional analysis in order to build a comprehensive network for multi-target multi-drug discovery (Figure 1).
